# Developing a national atlas to support the progressive control of tsetse-transmitted animal trypanosomosis in Zambia

**DOI:** 10.1186/s13071-025-07086-2

**Published:** 2025-11-10

**Authors:** Jackson Muyobela, Kalinga Chilongo, Milner Mukumbwali, Chris Sihoka, Massimo Paone, Giuliano Cecchi

**Affiliations:** 1 Department of Veterinary Services, Tsetse and Trypanosomosis Control Unit, Ministry of Fisheries and Livestock, Lusaka, Zambia; 2 Department of Zoology and Entomology, Forest and Agricultural Biotechnology Institute, University of Pretoria, Hatfield, Pretoria, South Africa; 3 Animal Production and Health Division, Food and Agriculture Organization of the United Nations (FAO), Rome, Italy

**Keywords:** African animal trypanosomosis, Database, Atlas, Geospatial, Zambia, *Glossina*

## Abstract

**Background:**

Tsetse flies (Diptera: Glossinidae) are the sole cyclical vectors of African trypanosomosis, a parasitic disease affecting both animals and humans. The national atlas of African animal trypanosomosis (AAT) and its tsetse vectors in Zambia is an initiative by the Tsetse and Trypanosomosis Control Unit (TTCU) within the Ministry of Fisheries and Livestock that aims to improve AAT surveillance and its progressive control by enhancing disease intelligence and data management.

**Methods:**

All field data collected by the TTCU from April 2009 to July 2025 were systematically assembled, georeferenced and harmonised. The data included entomological information on tsetse flies collected using mobile and stationary trapping devices, as well as animal trypanosomosis data obtained through the buffy coat technique (BCT).

**Results:**

Tsetse trapping was conducted in 3463 sites using mobile devices and in 478 locations using stationary traps. A total of 20,185 and 5189 flies were caught using the two data collection tools, respectively. Five species and subspecies of *Glossina* were detected: *G. morsitans morsitans* (65%), *G. m. centralis* (32%), *G. pallidipes* (2%), *G. fuscipes martinii* (0.8%) and *G. brevipalpis* (0.2%). As for AAT, 7652 animals (7348 cattle, 294 goats and 10 dogs) were tested in 148 locations. Of these, 329 animals (321 cattle, 7 goats and 1 dog) were found to be infected, with a prevalence of 4.37%. *Trypanosoma congolense* accounted for the highest number of infections (86%), with *Trypanosoma vivax* and *Trypanosoma brucei* representing 12% and 2% of the total infections, respectively.

**Conclusion:**

The national database of AAT and tsetse presented here established an effective information system to manage epidemiological data for the planning and monitoring of interventions against tsetse and trypanosomosis in Zambia. The atlas is planned to be regularly and promptly updated to ensure that current information is available to the TTCU, planners and other stakeholders involved in tsetse and AAT control.

**Graphical Abstract:**

**Figure:**
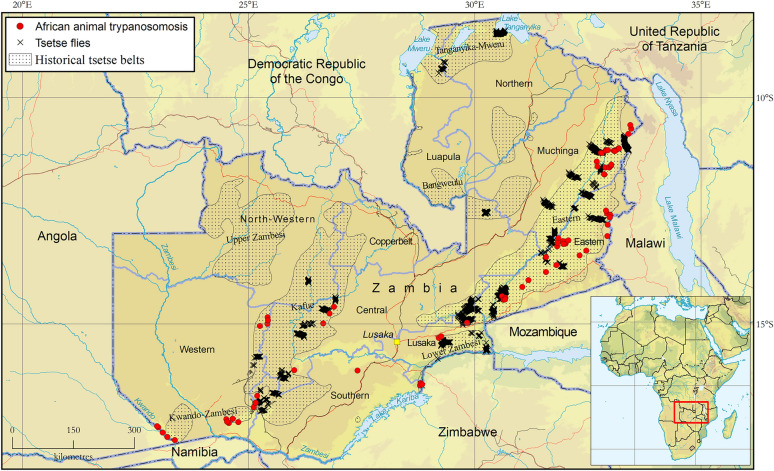

**Supplementary Information:**

The online version contains supplementary material available at 10.1186/s13071-025-07086-2.

## Background

Tsetse flies (Diptera: Glossinidae) are the sole cyclical vectors of African animal trypanosomosis (AAT; also known as nagana) and human African trypanosomiasis (HAT; also known as sleeping sickness) [1, 2]. Nagana refers to a variety of acute and/or chronic wasting diseases caused by unicellular protozoan parasites of the genus *Trypanosoma* (Kinetoplastida) that constrain most animal and mixed crop farming efforts in sub-Saharan Africa [3]. The main tsetse-transmitted pathogenic animal trypanosomes include *Trypanosoma* (*Trypanozoon*)* brucei* spp., *T.* (*Nannomonas*) *congolense*, *T.* (*Duttonella*) *vivax* and *T.* (*Nannomonas*) *simiae* [4, 5]. The non-tsetse fly-transmitted *Trypanosoma evansi* and *Trypanosoma equiperdum* cause the animal diseases surra and dourine, respectively [6], and it remains controversial whether they should be considered to be taxonomically distinct from *T. brucei* [7]. Where AAT is prevalent, meat, milk, animal manure and draft power production are significantly reduced. The disease causes morbidity and mortality in millions of cattle, despite the administration of over 35 million doses of trypanocidal drugs annually [8]. Therefore, AAT is a major constraint to improved livestock production and productivity in sub-Saharan Africa [9, 10].

In Zambia, 277,000 km^2^ of landmass was historically estimated to be infested with tsetse flies, occurring in seven tsetse belts [11]. Six species and subspecies of the tsetse fly have been identified in these belts, namely the riverine flies *Glossina fuscipes fuscipes* Newstead and *G. f. martinii* Zumpt; the forest fly *G. brevipalpis* Newstead; and the savannah flies *G. pallidipes* Austen and *G. morsitans*: *G. m. centralis* Machado and *G. m. morsitans* Westwood [12, 13]. *Glossina morsitans* is the most widely distributed species, covering the entire infested range of the country [11, 14]. The south-eastern part of the country is infested with *G. m. morsitans* (i.e. in the Luangwa-Lunsemfwa and Lower Zambezi tsetse belts). In contrast, *G. m. centralis* occurs in the north-western part of the country (in the Bangweulu, Kafue, Kwando-Zambezi, Tanganyika-Mweru and the Upper-Zambezi tsetse belts) [11]. The distribution of the other three species is sympatric with *G. morsitans,* but they have a much lesser geographic extent [11]. Given its high trypanosome vectorial capacity and wide distribution, *G. morsitans* is the major vector of both AAT and HAT in Zambia. *Glossina pallidipes* is also of high medical and economic importance and is associated with historical and current HAT foci [15]. Over 60% of Zambia’s cattle population is estimated to be at risk of animal trypanosomosis [16]. The average AAT prevalence ranges from 1% to 8.9% based on parasitological techniques [17, 18] and from 4% to 23.6% using molecular methods [18]. As such, AAT has been ranked the second most important livestock zoonotic disease in Zambia after anthrax [19].

The availability of accurate knowledge of tsetse distribution patterns is essential for gaining insights into trypanosomosis epidemiology [20] and the development of risk-based disease control strategies [5]. Several factors influence local tsetse distribution patterns, including climate [21], vegetation [22] and host availability [23]. Climate changes have been reported in sub-Saharan Africa over the last century [24], and habitat fragmentation, defined as the breakup of native vegetation into smaller isolated fragments [25], has resulted in the progressive loss of natural vegetation and the disappearance of large game animals due to anthropogenic activities [22]. These changes have resulted in notable modifications to tsetse habitats, leading to significant changes to tsetse distributions [26], especially for savannah species [23]. Changes in tsetse distribution have important implications for AAT disease epidemiology and must, therefore, be considered when designing control programmes. Thus, maintaining reliable information on past and current tsetse and disease distributions is a key component for rational tsetse and trypanosomosis control, especially in the framework of the progressive control pathway (PCP) approach [5].

Historical tsetse distribution maps in Zambia are more than 40 years old [12], with a recent update provided by the Food and Agriculture Organization of the United Nations (FAO) continental atlas of the distribution of tsetse flies in Africa [27]. However, total reliance of the FAO continental atlas on data published in scientific journals, its wide geographical gaps in Zambia and the exclusion of information published after 2020 significantly limit its usefulness at the national level [27]. Furthermore, country-wide AAT distribution maps have, to the best of our knowledge, never been produced for Zambia. Against this backdrop, a substantial amount of largely unpublished data on both tsetse and AAT has been collected in recent years, primarily by the specialised national structure mandated to control tsetse and AAT in Zambia [i.e. the Tsetse and Trypanosomosis Control Unit (TTCU)]. However, despite the availability of these data, a unified, centralised and regularly updated database is lacking. This gap hinders evidence-based decision-making, while the importance of accurate epidemiological mapping in the implementation of risk-based approaches to disease management cannot be overstated. Therefore, the successful rollout of an AAT control strategy based on PCP principles at the national level in Zambia hinges on the development of a nationwide spatially explicit information system on AAT and its vectors (i.e. an atlas). The objective of this study was to develop such a tool.

## Methods

The national atlas for tsetse and AAT for Zambia was developed using the general principles outlined by the FAO for the continental atlas [27, 28]. However, while the continental atlas relies solely on peer-reviewed scientific publications, the present national atlas consists of all the data that was collected by the TTCU between April 2009 and July 2025, including both published [14, 17, 29] and unpublished data (field records kept by the TTCU). Furthermore, several AAT-endemic countries have already established similar national-level information systems [30–35], and their work provided additional methodological references for developing the national atlas for Zambia.

### Input data

#### Tsetse data

Tsetse trapping data consisted of fly catches based on various stationary and mobile trapping devices. Among the stationary traps, the Epsilon trap was the most widely used [36], being critical in the sampling of *G. pallidipes*, *G. m. centralis*, *G. m. morsitans,* and, to a lesser extent, *G. brevipalpis*. Epsilon traps were baited with methyl ethyl ketone (MEK) and 1-octen-3-ol attractants, and dispensed according to methods described by Torr et al. [37]. Briefly, a 500-ml glass bottle with a 2-mm aperture in the stopper was used to dispense MEK at a rate of 150 mg/h, while polyethylene sachets of 4 × 5-cm 500 gauge/0.125 mm dispensed 3 g 1-octen-3-ol at 0.5 mg/h. Un-baited small sticky traps comprising blue (polyester; PermaNet, Vestergaard Frandsen, Copenhagen K, Denmark) and black (100% black, 225 g/m^2^ polyester, Q15093; Sunflag, Nairobi, Kenya) panels, each 0.25 × 0.25 m in size, and a transparent sticky film (Luminos 4 adhesive rolls; Rentokil Initial, Liverpool, UK) placed on top to facilitate the capture and enumeration of landing flies [38] were used to sample *G. fuscipes*. Data from newly developed large, baited sticky traps of various sizes (unpublished data) have also been included in the atlas. All stationary traps were deployed for 1 trapping day.

Regarding mobile trapping systems, two different types were used to capture tsetse. Firstly, black screen fly rounds (BFR), consisting of a catching party of two individuals with hand nets and a baited 1 × 1.5-m black screen [39], were used to sample populations of *G. m. centralis* and *G. m. morsitans*. The sampling procedure was such that the catching party walked along a predetermined transect at normal speed, stopping for 3 min at 200-m intervals to capture tsetse flies that had landed on the screen and each other [40]. The use of BFR in sampling these tsetse is supported by the observation that movement increases the attractiveness of an object to tsetse species [41]. Vale [42] also demonstrated that mobile baits are more effective in trapping *G. m. morsitans* than stationary baits. Secondly, a baited two-sided, all-blue (blue polyester; ParmaNet, Vestergaard Frandsen) vehicle-mounted sticky trap (VST) with one-sided adhesive film (FE45; Rentokil Initial) [14, 43] was used to sample *G. m. centralis* and *G. m. morsitans.* The VST traversed selected transects at a maximum speed of 20 km/h, making interval stops each 1 km to collect captured flies [43]. A significant advantage of the VST over BFR is that it is rapid and independent of operator skill, and requires significantly less labour to sample a larger geographic area [14].

For all trapping devices, captured flies were identified to the species level, sexed and enumerated, and the geographic coordinates of the capture site were recorded using a handheld global positioning system (GPS). Species identification was done morphologically using keys provided by Leak et al. [39]. By contrast, subspecies determination was based on the location of the survey using historical distribution maps [12].

Field data were captured using two data sheet types, namely mobile and stationary data collection sheets, which were adapted from Robinson [40] and Leak et al. [39] (Additional File 1: Data sheets). Briefly, the data sheets capture information such as tsetse belt, administrative units, geographic coordinates, date of survey, duration of trapping, number of flies captured, species and sex of the trapped tsetse. Data were collected during pre-intervention surveys (baseline) and intervention monitoring (during the study and post-intervention). A significant proportion of VST data was collected during a nationwide survey aimed at updating knowledge on the distribution of *G. morsitans* in Zambia and enhancing ecological niche modelling [14]. Finally, targeted surveys were conducted in several localities to bridge the information gap that became apparent during atlas building.

#### Animal trypanosomosis data

The buffy coat technique (BCT) [44], a parasitological concentration method that requires fresh blood, was used to detect all AAT cases captured in the atlas. For tsetse, data on AAT were collected during baseline surveys and AAT monitoring in areas subjected to tsetse control operations. Sampling was conducted by assembling livestock from a given area at one site and randomly selecting animals for screening. Blood samples were collected from the ear vein of each animal into heparinised micro-haematocrit capillary tubes, which were then centrifuged for 5 min at 12,000 rpm. After centrifugation, the packed cell volume (PCV) was determined per sample animal. The buffy coat was examined microscopically at ×400 magnification, and trypanosome species determination was based on the distinct characteristic movement of trypanosomes in blood [45]. On wet films, *T. brucei* exhibits bihelical or “snake-like” motion between red blood cells, while *T. vivax* is characterised by its rapid, whirling movement across the microscopic field. The movement of *T. congolense* is relatively slower and more sluggish, with the parasite appearing to be attached to red blood cells. Data were captured using AAT survey/surveillance record sheets which include the following information: animal identity, geographic coordinates, administrative units of the survey site, date of the survey, name of animal owner, number of animals presented and sampled, number of animals that tested positive for trypanosomosis (by trypanosome species), PCV and age of the animal (Additional File 2: Data sheets). The names of the owners of the animals are, however, not included in the database.

### Structure of the atlas

Similar to the previous continental and national atlases, the atlas for Zambia was structured into a data repository and a database. In the data repository, the source [in both PDF and Microsoft (Microsoft Corp., Redmond, WA, USA) Excel formats] and the processed/cleaned files used as input for the database were included. Source files that provided data for the atlas were represented by scanned PDF copies of field record sheets, stored separately for tsetse and AAT. Spreadsheet versions of these source files (Microsoft Excel) were also stored in the repository, without data editing and/or processing. Processed/cleaned files (i.e. data-verified files that capture all the information relevant for the database and are Geographic Information System (GIS)-ready) were also stored in the repository. The repository currently includes 34 field record sheets for AAT and 34 for tsetse.

The database consists of three Microsoft Excel spreadsheets, one for tsetse stationary traps, one for tsetse mobile trapping devices and one for AAT. A detailed description of the content of each column within each Excel spreadsheet is given in Additional File 3: Database and Additional file 4: Database. Both tsetse databases include source information (i.e. the data record sheet in the repository), the geographical information about the trapping sites [administrative units, tsetse belt and geographic coordinates (latitude and longitude in decimal degrees)], the type of trap or mobile device, odour attractant used, the period of the survey, tsetse species (including number of flies caught, sex, and apparent density). The apparent density (AD) of stationary traps was computed as described by Leak et al. [39], where the number of flies caught is divided by the number of traps and trapping days (i.e. flies per trap per day). To make a uniform data structure for mobile sampling devices, all BFR catches collected at 200-m stops were amalgamated to 1-km intervals and allocated the mid coordinate of that kilometre [43]. Computation of an AD for mobile sampling devices followed the approach applied by Muyobela et al. [14]. Briefly, trap catches in a 1-km transect were interpreted as a response from a 1 × 1-km grid, an interpretation that accounts for the daily displacement of *G. morsitans* (estimated to be between 167 m and 1.3 km [46]), a key factor in making tsetse available for capture. Since the time of exposure of a sampling device within a stated distance is part of the sampling effort, computation of AD also accounted for the length of time it was operated in the 1-km transect. Therefore, the AD for mobile traps was reported as the number of flies per kilometre^2^ per unit time [14].

Like the tsetse database, the AAT database includes information on the survey/monitoring sites (administrative units, location name and geographic coordinates), diagnostic method, the survey period, sample size and the results of the survey in terms of the number and prevalence of trypanosome infections (disaggregated by trypanosome species). Cattle, goats and dogs were the animal species included, and information on the breed, sex and age of the animal was also captured.

### Atlas development process

The initiative to develop the atlas was launched in 2021 with the appointment of three national-level focal persons in charge of data management and mapping. The standard procedure for atlas development involving data collation (i.e. repository building), data processing and database building [28] was followed. To build the repository, existing AAT and tsetse field data available at the TTCU national office were collated and assessed. In the current atlas, data source selection criteria included the availability of geographic coordinates of sample sites, species identification of parasites detected and identification of tsetse flies captured. Upon confirming that a particular data source met the inclusion criteria, the hard copy of the data source was scanned (PDF), digitised (as a Microsoft Excel file) and stored in the data repository. Next, each data source (Microsoft Excel file) was checked, cleaned, verified, harmonised, geo-referenced, formatted and completed. Quantum GIS version 3.14 was used to verify the accuracy of the GPS coordinates recorded in the field, and the format of various attribute values was standardised. Where some information items were not explicitly captured in the original data sheet (e.g. tsetse apparent densities when only the number of fly catches was reported), data completion was undertaken. Finally, the databases were built by amalgamating processed source data into single Excel files from which required data/information can be retrieved/extracted, including in the form of maps. FAO developed the maps for this paper using ArcMap 10.5 GIS software.

## Results

### Tsetse distribution

**Fig. 1:**
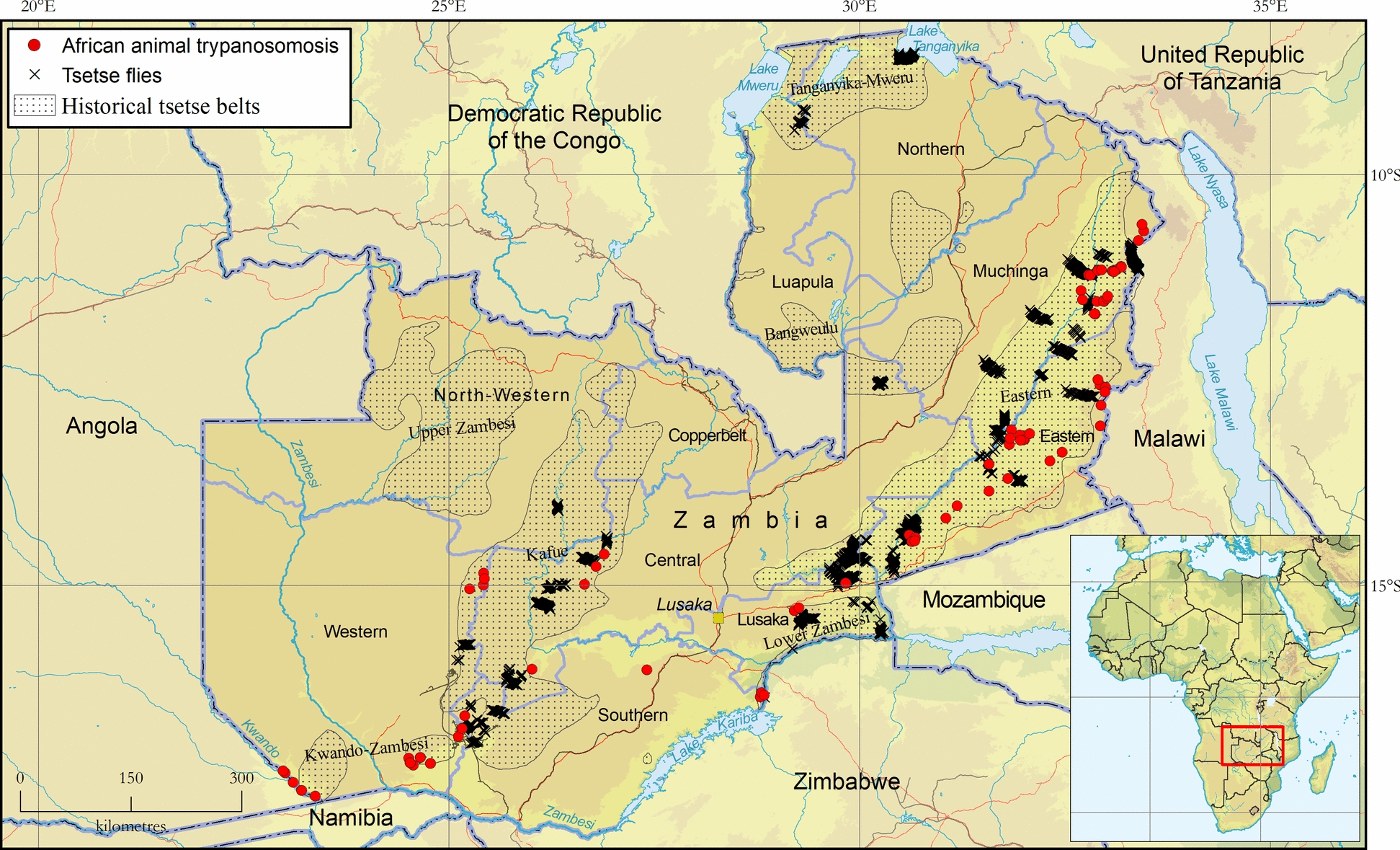
Presence of tsetse flies (genus* Glossina*) (black crosses) and African animal trypanosomosis (red dots) in Zambia. Data collection period: April 2009 to June 2025. Source: Tsetse and Trypanosomosis Control Unit (TTCU). Data available in Additional file 5: Dataset, Additional file 6: Dataset and Additional file 7: Dataset

A total of 24,577 tsetse were captured from both mobile and stationary traps. Mobile traps captured 20,185 (82%) flies while stationary traps recorded 5189 (18%). Overall, the catch composition consisted of *G. m. morsitans* (65%), *G. m. centralis* (32%), *G. pallidipes* (2%), *G. f. martinii* (0.8%) and *G. brevipalpis* (0.2%). *Glossina f. fuscipes* was not detected. Regarding mobile traps, 3475 km were traversed, covering parts of all tsetse belts (Table 1). Five of the seven historical tsetse belts recorded catches (i.e. all except for the Kwando-Zambezi and Upper-Zambezi belts) (Fig. 1). The Luangwa-Lunsemfwa tsetse belt was the most extensively surveyed (1986 km covered), while the highest recorded tsetse apparent density of 1.6 flies per km^2^ per min was recorded in the Tanganyika-Mweru tsetse belt (Table 1). As shown in Table 2, the VST traversed a greater distance and captured more flies than the BFR, despite the former having been operated for only 5 years (2019–2025) while the latter was in operation over the 16-year period during which all the data in the atlas were collected (2009–2025).

**Table 1:** Tsetse fly catches from mobile sampling devices

Tsetse belt	Distance (km)	*Glossina m. morsitans* (*n*)	*Glossina m. centralis* (*n*)	*Glossina pallidipes* (*n*)	*Glossina brevipalpis* (*n*)	*Glossina f. martinii* (*n*)	Total catch (*n*)	Average AD
Bangweulu	121	0	306	0	0	0	306	0.25
Kafue	489	0	4512	0	0	0	4512	0.87
Kwando-Zambezi	206	0	0	0	0	0	0	0.00
Lower-Zambezi	270	2560	0	92	0	0	2652	0.90
Luangwa-Lunsemfwa	19,86	10,320	0	489	0	0	10,809	0.36
Tanganyika-Mweru	119	0	18,95	0	11	0	1906	1.60
Upper Zambezi	284	0	0	0	0	0	0	0.00
*Total*	3475	12,880	6713	581	11	0	20,185	

*AD* Apparent density (number of tsetse flies per km^2^ per min)

**Table 2:** Comparison of tsetse fly catches from mobile sampling devices

Mobile device	Start year	End year	Distance (km)	*Glossina m. morsitans* (*n*)	*Glossina m. centralis* (*n*)	*Glossina pallidipes* (*n*)	*Glossina brevipalpis* (*n*)	*Glossina f. martinii* (*n*)	Total (*n*)
BFR	2009	2021	1483	4688	368	228	0	0	6327
VST	2019	2025	1897	6933	6345	202	11	0	13,858
*Total*			3380	11,621	6713	430	11	0	20,185

*BFR* Black screen fly round, *VST* vehicle-mounted sticky trap

In the case of the stationary traps, 478 trapping events were included in the database. The trapping events were undertaken in six of the seven tsetse belts, with tsetse fly catches recorded in four of these (Table 3). Apparent density was highest in the Luangwa-Lunsemfwa and Kafue tsetse belts. Data on the catch by the large sticky traps makes up the larger proportion (3161 tsetse fly catches; 61%) of catches from stationary traps, with the catch by Epsilon Trap (1966 tsetse fly catches) and Tiny Sticky Traps [62] making up 38% and 1%, respectively.

**Table 3:** Tsetse fly catches from stationary traps

Tsetse belt	No. of traps	*Glossina m. morsitans* (*n*)	*Glossina m. centralis* (*n*)	*Glossina pallidipes* (*n*)	*Glossina brevipalpis* (*n*)	*Glossina f. martinii* (*n*)	Total (*n*)	Average AD
Bangweulu	30	0	0	0	0	62	62	2.07
Kafue	139	0	1070	0	0	0	1070	7.70
Kwando-Zambezi	25	0	0	0	0	0	0	0.00
Lower-Zambezi	63	4	0	1	0	0	5	0.08
Luangwa-Lunsemfwa	207	3251	0	796	6	0	4053	15.62
Upper Zambezi	14	0	0	0	0	0	0	0.00
*Total*	478	3255	1070	796	0	62	5189	10.86

*AD* Apparent density (number of tsetse flies per trap per day)

**Fig. 2:**
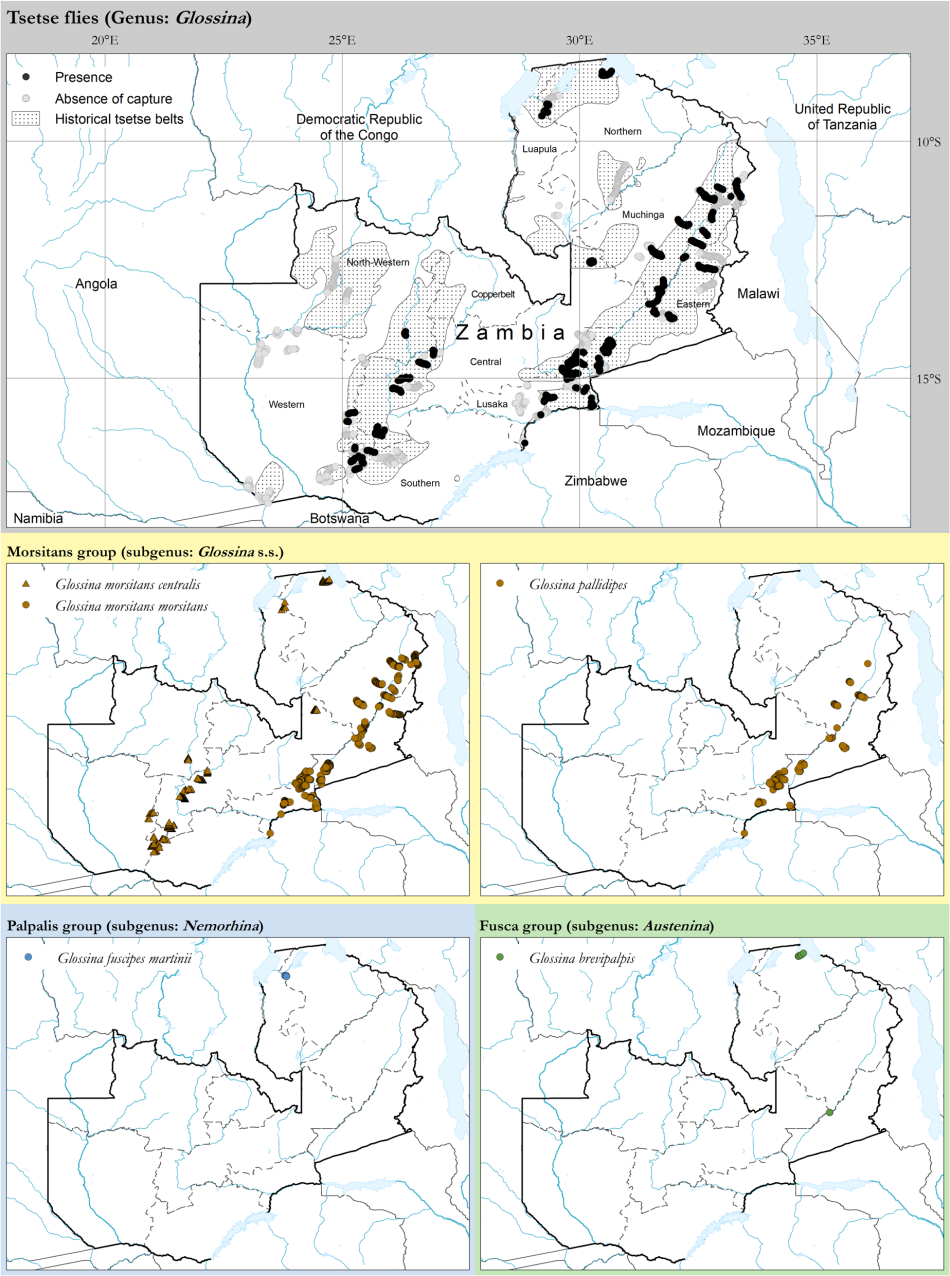
Presence of tsetse fly species in Zambia (coloured circles and triangles). Data collection period: April 2009 to June 2025. Source: Tsetse and Trypanosomosis Control Unit (TTCU). Data are available in Additional file 5: Dataset and Additional file 6: Dataset

As shown in Fig. 2, *G. m. centralis* and *G. m. morsitans* were confirmed to have the widest geographical distribution, followed by *G. pallidipes*. The riverine *G. f. martinii* was recorded only in the Tanganyika Mweru tsetse belt, and the forest *G. brevipalpis* was detected in the Luangwa-Lunsemfwa and Tanganyika Mweru tsetse belts.

### Bovine trypanosomosis

**Fig. 3:**
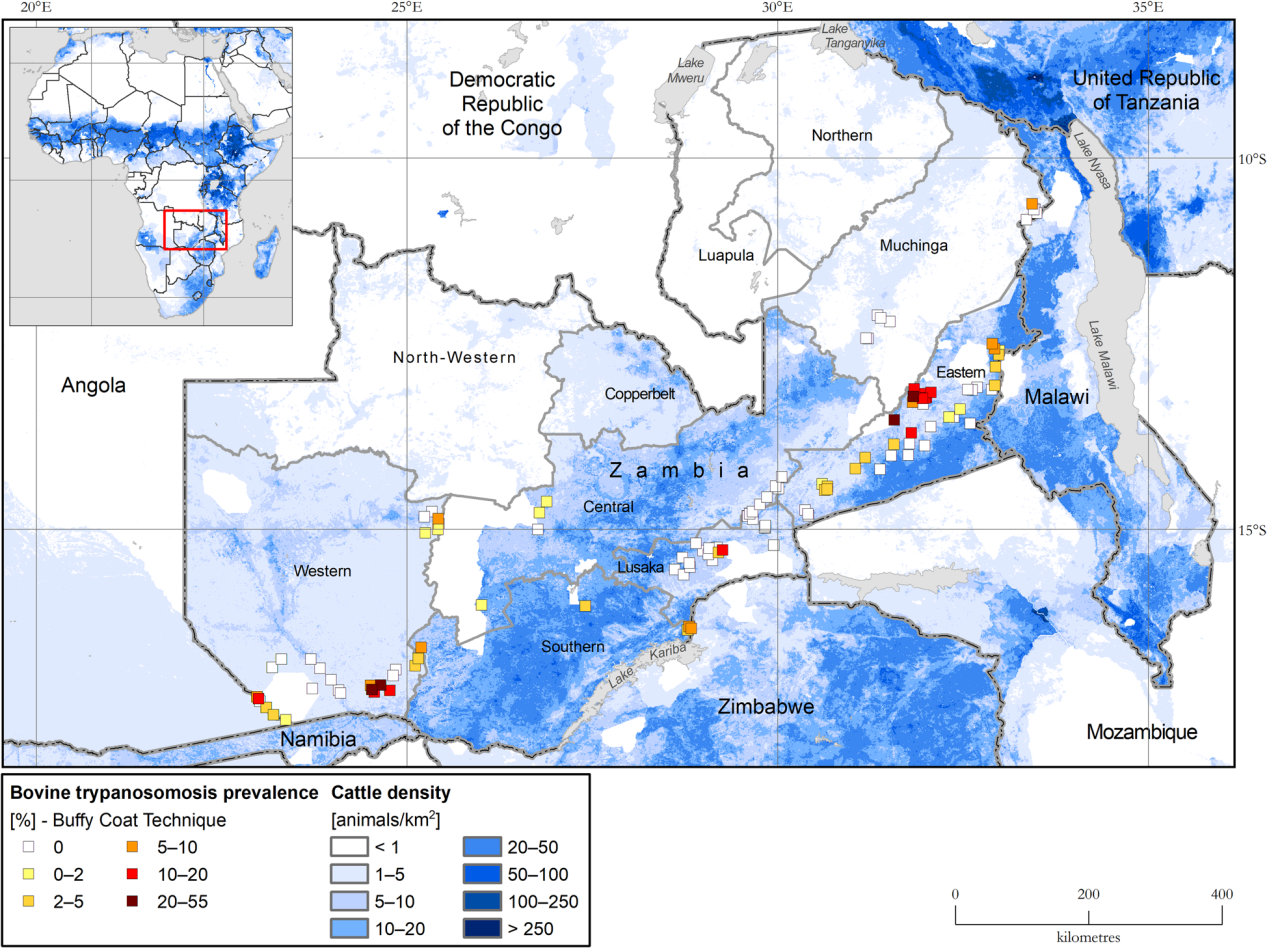
Bovine trypanosomosis prevalence in Zambia as determined with the buffy-coat technique. Trypanosomosis data collection period: April 2009 to June 2025. Bovine trypanosomosis data source: Tsetse and Trypanosomosis Control Unit (TTCU), Zambia. Cattle density data source: FAO Gridded Livestock of the World [47]. Data are available in Additional file 7: Dataset

**Fig. 4:**
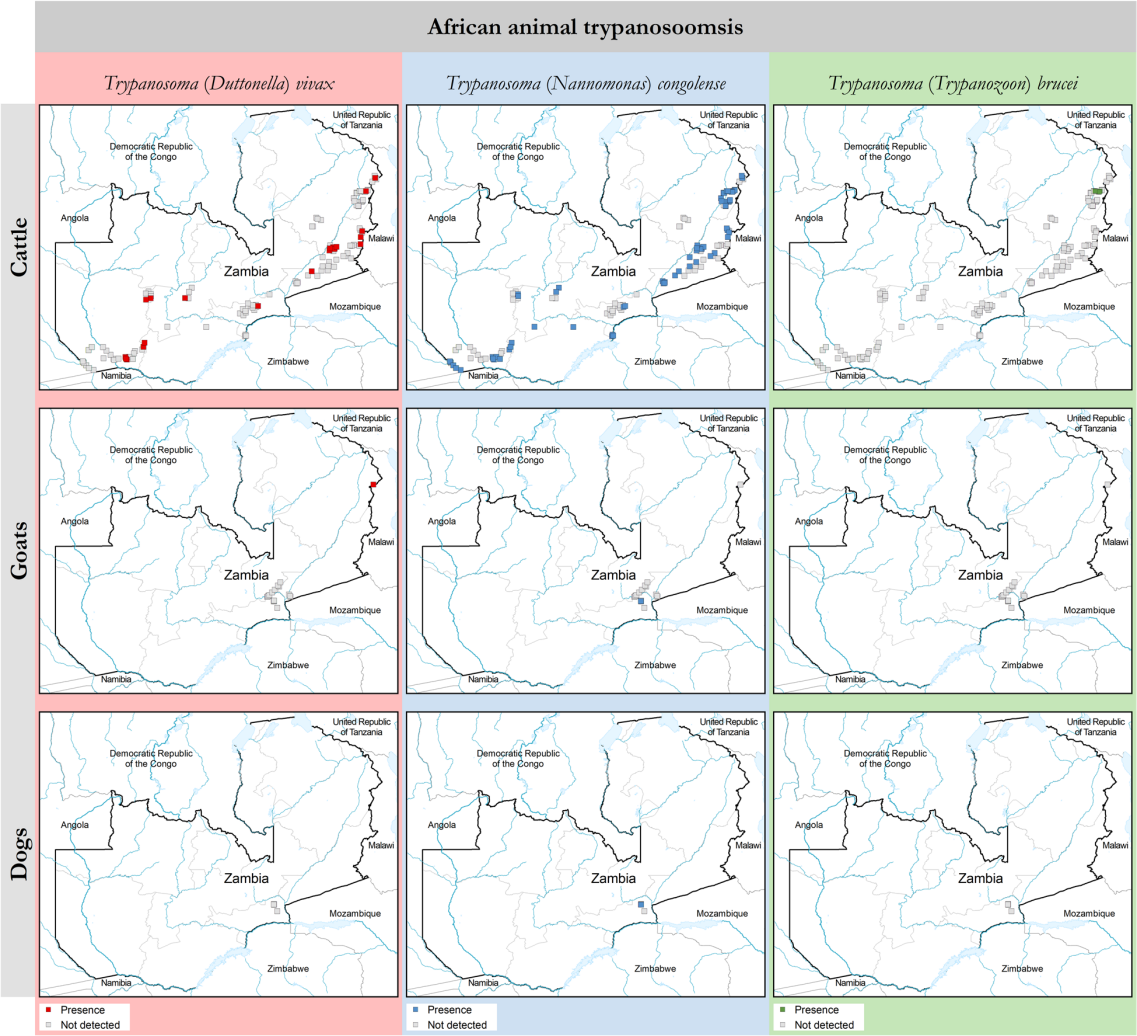
Presence (coloured squares) and absence (surveyed but not detected, grey squares) of *Trypanosoma vivax* and *Trypanosoma congolense* in cattle, goats and dogs as determined with the buffy coat technique. Trypanosomosis data collection period: April 2009 to June 2025. Source: Tsetse and Trypanosomosis Control Unit (TTCU), Zambia. Data are available in Additional file 7: Dataset

The bovine trypanosomosis component of the database comprises 205 parasitological records collected from 148 distinct locations in four tsetse belts (Table 4). The sampling intensity was 7348 cattle, with 321 infections detected (277*T. congolense*, 32* T. vivax* and four *T. brucei*). The observed geographic occurrence of bovine trypanosomosis is shown in Fig. 1, which also indicates that most infections were detected in the Kafue, Luangwa-Lunsemfwa and Kwando-Zambezi tsetse belts. The highest prevalence of bovine trypanosomosis was recorded in the Luangwa-Lunsemfwa tsetse belt (Table 4). The prevalence of *T. congolense* (3.37%) was higher than that of *T. vivax* (0.47%). The prevalence of bovine trypanosomosis over the sampled locations ranged from zero to 55% (Fig. 3), and 84% of locations reported the use of the trypanocidal drugs (i.e. diminazene aceturate and/or isometamidium chloride). The data included in the database could not confirm the presence or absence of bovine trypanosomosis in the other tsetse belts (i.e. Bangweulu, Upper Zambezi and Tanganyika-Mweru), as surveys were not conducted in these areas. Sampling for AAT was only conducted in tsetse belts associated with a significant cattle population (Fig. 3). Figure 4 shows the distribution of trypanosome species as detected during AAT sampling and highlights that *T. vivax* and *T. congolense* had a similar distribution, with both being detected in the Kafue, Luangwa-Lunsemfwa and Kwando-Zambezi tsetse belts; however, *T. congolense* affected significantly more locations than *T. vivax*. *Trypanosoma brucei* infections were only detected in the Luangwa-Lunsemfwa tsetse belt.

**Table 4:** Prevalence of bovine trypanosomosis as determined by the buffy coat technique

Tsetse belt	Mean PCV of uninfected bovines (%)	Mean PCV of infected bovines (%)	No. of locations	Bovine sample size (*n*)	No. of *Trypanosoma vivax* detected	No. of *Trypanosoma congolense* detected	No. of *Trypanosoma brucei* detected	Total infections detected (*n*)	Prevalence (%)
Kafue	33	30	11	783	3	9	0	12	1.58
Kwando-Zambezi	31	28	26	2457	12	85	0	97	3.30
Lower-Zambezi	33	25	17	824	2	16	0	18	2.27
Luangwa-Lunsemfwa	33	26	94	3288	23	167	4	194	9.41
*Total*			148	7348	40	277	4	321	4.37

*PCV* Packed Cell Volume

### Caprine and canine trypanosomosis

The AAT database comprises four and 20 parasitological records of canine and caprine trypanosomosis, respectively, recorded from the Luangwa-Lunsemfwa tsetse belt. The sampling intensity consisted of 294 goats and 10 dogs, from which seven goats (6 *T. vivax* and 1 *T. congolense*) and one dog (*T. congolense*) were found to be infected. The spatial occurrence of caprine and canine trypanosomosis is shown in Fig. 4.

## Discussion

This paper presents the first edition of the national atlas of AAT and its tsetse vectors in Zambia, an initiative implemented by the TTCU and technically supported by the FAO. The atlas includes TTCU data collected over 16 years (April 2009 to July 2025) and represents the largest database of tsetse distribution ever assembled to date in Zambia. This atlas is also the first attempt to collate and map AAT data at the national level. The atlas is a simple, flexible and dynamic information system that lends itself to future enhancements and updates, thus enabling a more efficient planning and execution of both survey/surveillance and control operations.

Currently, the atlas data confirm the presence of tsetse in all but two of the historical tsetse belts in Zambia. In the Kwando-Zambezi Belt, the observed absence of tsetse flies is attributed to two sequential aerial spraying operations implemented in the tsetse belt in 2009 and 2014, which are likely to have achieved lasting elimination. These operations were a continuation of the regional aerial tsetse eradication programme that started in 2001 in Botswana [48–50] and culminated in two separate operations in Zambia. The data presented here clearly shows that no tsetse has been captured in the Kwando-Zambezi Tsetse in Zambia Belt area since the implementation of these operations.

In the Upper-Zambezi belt, the absence of tsetse could be attributed to limited sampling effort, as data included for this belt originate from a single VST survey conducted in November 2021 [14]. While the effectiveness of the VST has been confirmed both experimentally [43] and under field conditions [14], a single survey does not provide sufficient data to enable the conclusion to be drawn that tsetse is absent in this belt. We therefore envisage follow-up surveys using VST and other sampling tools to improve our confidence in the absence of tsetse in this area. This is especially important as the area still has large game animals and is earmarked for cattle stocking.

Of the six known tsetse species and subspecies that occur in Zambia [11–13], *G. m. morsitans*, *G. m. centralis*, *G. pallidipes*, *G. brevipalpis* and *G. f. martinii* have been captured in the database; *G. f. fuscipes* was, however, not detected. In Zambia, this tsetse fly has been reported to occur on the shores of Lake Tanganyika [12], and its absence in the data is attributed to the fact that sampling on these shores was not carried out. While species identification was easily achieved using the keys provided by Leak et al. [39], subspecies identification was based on geographic distribution [12]. It would, therefore, be of significant benefit if, in the future, studies were undertaken to verify tsetse subspecies identity using methods based on both dissection [39] and molecular tools [51]. Regardless of this shortcoming, our results corroborate the findings of other researchers [11, 12, 14] that the subspecies of *G. morsitans, G. m. centralis* and *G. m. morsitans* are the most widespread tsetse species in Zambia, followed by *G. pallidipes*. An interesting result was the observation of *G. brevipalpis* in the Tanganyika-Mweru, which, to the best of our knowledge, is the first report of its presence in this belt since 1952. Maseko [52] previously reported the presence of this species only in the Luangwa-Lunsemfwa belt, with the highest number of flies caught using artificial refuges. Surveys conducted in the Luangwa-Lunsemfwa tsetse belt between April 2009 and July 2025 confirmed the presence of *G. brevipalpis* in this belt.

Quantitatively, the data presented here indicate that mobile sampling enabled us to cover a much larger geographical area than the use of stationary traps. This could be attributed to the fact that mobile tools can be rapidly deployed over large areas and easily detect the most prevalent tsetse, *G. morsitans,* than the use of stationary traps [39, 42, 43]. Of key note were results obtained from the VST, which, for the first time since 1952, enabled the sampling of all tsetse belts in Zambia, and this was done within 1 month [14]. These results highlight the utility of the VST for rapid, large-scale tsetse sampling, and we therefore recommend its use where *G. morsitans* and related species occur.

All three of the known tsetse-transmitted trypanosomes that infect cattle, *T. congolense*, *T. vivax* and *T. brucei*, were detected and captured in the database. However, the number of detected *T. brucei* infections was very low. Higher numbers of *T. brucei* infections in cattle have, however, been previously reported in Zambia [53–56]. The low number of *T. brucei* cases in our surveys could be attributed to the relatively low capacity of parasitological methods, such as BCT, to detect low parasitaemia associated with *T. brucei* infections, especially in the chronic phase of the infection [56–58].

Our data show that AAT sampling mainly covered parts of only three tsetse belts: the Luangwa-Lunsemfwa, Kwando-Zambezi and Kafue tsetse belts. This apparent focus is in part attributed to the concentration of the country’s cattle population in areas affected by the three tsetse belts [47, 59], and the focus of AAT surveillance activities reflects cattle distribution. However, in light of the Government's plans to stock non-traditional cattle-rearing communities located in the north of the country [60], it is crucial that, in the future, surveys should also be undertaken in these areas to quantify the risk of AAT. The availability of goats and dogs in these areas provides an opportunity to estimate AAT prevalence in these animals and to use the findings to evaluate the risk if cattle were introduced.

The overall prevalence of AAT among sample locations was found to be 4.37%, a value consistent with that reported by Machila et al. [61]. This prevalence is higher than national estimates obtained with the same diagnostic method in Kenya [30], but lower than that estimated in Ethiopia [33], Burkina Faso [34] and Mali [35]. AAT prevalence was observed to be highly variable between sampling locations, ranging from zero to 55%. Variability in AAT parasitological prevalence due to location has been previously reported [61] and is reflected in the different prevalence estimates that are reported in Zambia [17, 18, 56, 62]. This variability reflects differences in the risk of AAT infection among the sample sites. Dicko et al. [20] reported that entomological inoculation rate, environmental factors determining tsetse apparent densities and fly infection rates were important factors in predicting AAT risk. We therefore recommend studies to assess and quantify these parameters empirically and evaluate the variation in the risk of AAT infection in Zambia. Such an evaluation would provide helpful information to guide decision-making in the control of AAT.

**Fig. 5:**
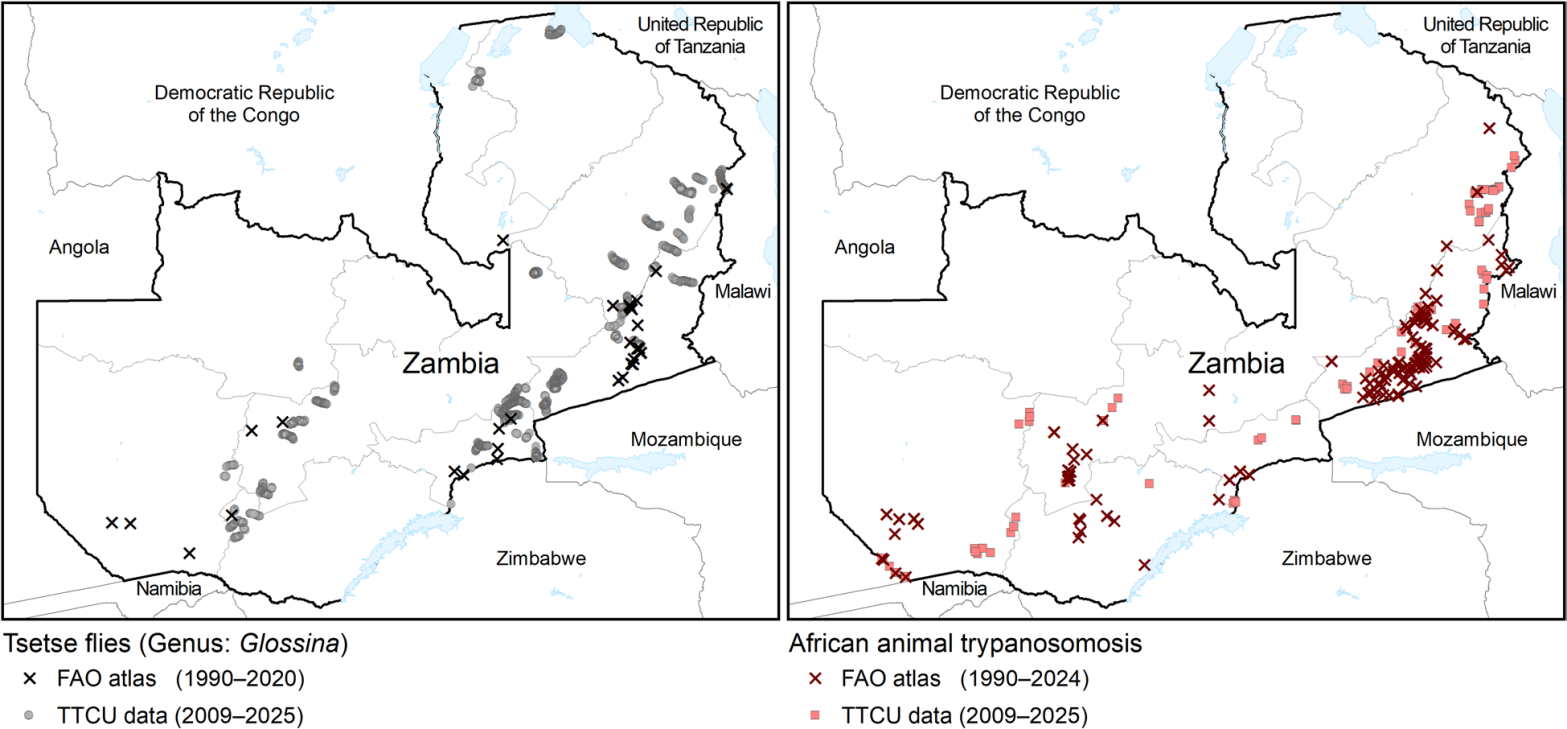
Comparison of the distribution pattern of African animal trypanosomosis and the tsetse fly in Zambia based on the FAO continental and Zambia atlases. FAO, Food and Agriculture Organization of the United Nations; TTCU, Tsetse and Trypanosomosis Control Unit

Comparison of the FAO and Zambia atlases shows a general agreement in the distribution pattern of AAT and tsetse in Zambia (Fig. 5). However, since the FAO atlas includes data collected over a longer period (1990–2020), it depicts a wider distribution of AAT than does the Zambia atlas. This suggests that there is an opportunity for additional data from other stakeholders to be included in the Zambia atlas, and this would enable a more accurate analysis of spatial trends over time. Regarding tsetse data, the Zambia atlas has substantially more data and a wider geographic coverage than the FAO atlas; it therefore provides a more accurate depiction of the tsetse distribution in the country, allowing for substantial improvements in future editions of the continental atlas.

We note that to support decision-making at the national level, the data on Zambia which the FAO extracted from publications for the continental atlas were made available to TTCU and, therefore, this effort did not need to be duplicated at the TTCU level. The approach of focusing the first edition of a national atlas only on data from the specialised national structure in charge of tsetse and AAT control has already been used by several countries, such as Ethiopia [33], Kenya [30] and Zimbabwe [31]; for Zambia and for all these other countries, one of the priorities for the second edition of the atlas would be to contact the authors of the scientific publications to gather and include in the atlas the related raw data, with the FAO-extracted data to be used only as a fallback option.

In addition to the gaps in the published information, the limitations of this first edition of the national atlas of Zambia include the use of only one diagnostic technique for AAT, biased sampling of tsetse belts with high cattle populations and the inclusion of data from only April 2009 and June 2025, which undoubtedly affected the distribution and prevalence estimates presented here.

## Conclusions

The work presented here highlights the importance of having an effective information system to manage epidemiological data for planning and monitoring of interventions against tsetse and trypanosomosis. The atlas must be regularly and promptly updated to ensure that the available information is always current, effectively guiding the planning and implementation of tsetse and AAT control. Future efforts aimed at improving the atlas should focus on capacity building in field data collection, including enhancement of the data recording sheet design and data management [30].

Following the example of FAO and its continental atlas [27], an open data policy was adopted for the present national atlas, with detailed datasets available as supplementary information (see Additional files). This is a first for national atlases of tsetse and animal trypanosomosis, and it answers increasing calls to enhance data findability, accessibility, interoperability and reuse for greater impact [63]. It is envisaged that this approach will be adopted in the future by an increasing number of countries, starting from those that developed or updated atlases within the framework of the COMBAT project [10].

## Supplementary Information

**Additional file 1: Record sheets.** Tsetse fly field records.

**Additional file 2: Record sheets.** Animal African trypanosomiasis field records.

**Additional file 3: Database. **Tsetse geospatial database information guide.

**Additional file 4: Database.** Animal African trypanosomiasis geospatial database information guide.

**Additional file 5: Dataset. **Dataset of the national atlas of tsetse flies in Zambia—mobile devices.

**Additional file 6: Dataset.** Dataset of the national atlas of tsetse flies in Zambia—stationary devices.

**Additional file 7: Dataset.** Dataset of the national atlas of animal trypanosomosis in Zambia.

## Data Availability

Data supporting the main conclusions of this study are included in the manuscript.

## Abbreviations

AAT: African animal trypanosomosis
AD: Apparent density
BCT: Buffy coat technique
BFR: Black screen fly round
FAO: Food and Agriculture Organization of the United Nations
GPS: Global positioning system
HAT: Human African trypanosomiasis
MEK: Methyl ethyl ketone
PCP: Progressive control pathway
PCV: Packed cell volume
TTCU: Tsetse and Trypanosomosis Control Unit
VST: Vehicle-mounted sticky trap
