# Supplementary material for: Developing a national atlas to support the progressive control of tsetse-transmitted animal trypanosomosis in Zambia
Source: Parasit Vectors. 2025 Nov 10;18:452. doi: 10.1186/s13071-025-07086-2 (PMC12604332; doi:10.1186/s13071-025-07086-2)
Supplement: Supplementary file 2 — Additional file 2: Record sheets. Animal African trypanosomiasis field records. [file 13071_2025_7086_MOESM2_ESM.docx]

**Trypanosomosis Survey Farmer Questionnaire**

Date: _____________________ Province: ______________________ District: _____________________ Village/Crush pen: __________________

Ward: _________________________ Vet camp: ___________________ Crush pen ID: _________________ Type of Survey: ____________________

Recorder: ______________________ Tsetse Intervention: _____________ Sample frame: * ____________ Tsetse or Trypanosomosis History: ** ___

Longitude: ____________________°E Latitude: _____________________°S Altitude: ___________________m

| Farmer’s Name | Village | No. of Animals Presented | Diminazene Aceturate  Y/N | If Y when last used | Isometamidium Chloride  Y/N | If Y when last used | No. Sampled | No. Positive | If positive, describe where animals are grazed |
| --- | --- | --- | --- | --- | --- | --- | --- | --- | --- |
|  |  |  |  |  |  |  |  |  |  |
|  |  |  |  |  |  |  |  |  |  |
|  |  |  |  |  |  |  |  |  |  |
|  |  |  |  |  |  |  |  |  |  |
|  |  |  |  |  |  |  |  |  |  |
|  |  |  |  |  |  |  |  |  |  |
|  |  |  |  |  |  |  |  |  |  |
|  |  |  |  |  |  |  |  |  |  |
|  |  |  |  |  |  |  |  |  |  |
|  |  |  |  |  |  |  |  |  |  |
|  |  |  |  |  |  |  |  |  |  |
|  |  |  |  |  |  |  |  |  |  |
|  |  |  |  |  |  |  |  |  |  |
|  |  |  |  |  |  |  |  |  |  |
|  |  |  |  |  |  |  |  |  |  |
|  |  |  |  |  |  |  |  |  |  |
|  |  |  |  |  |  |  |  |  |  |
|  |  |  |  |  |  |  |  |  |  |
|  |  |  |  |  |  |  |  |  |  |

***Sample frame**: Commercial farm (1); Crushpen/Cattle group/Collection of Villages (2); Individual village (3)

****Tsetse or Trypanosomosis History**: No history of tsetse or trypanosomosis in over 6 years – 0; No history of tsetse or trypanosomosis in over 5 years – 1; No history of tsetse or trypanosomosis in over 4 years – 2; No history of tsetse or trypanosomosis in over 3 years – 3; No history of tsetse or trypanosomosis in over 2 years – 4; No history of tsetse or trypanosomosis in over 1 year – 5; Disease currently present – 6.

Sheet __ of__

**Trypanosomosis Survey/Surveillance Record Sheet**

Date: _____________________ Province: ______________________ District: _____________________ Village/Crush pen: ______________

Ward: _________________________ Vet camp: ___________________ Crush pen ID: _________________ Type of Survey: ________________

Recorder: ______________________ Team members __________________________________________________ Tsetse Intervention: _____________

Longitude: ____________________°E Latitude: _____________________°S Altitude: ___________________m

| Sample No | Farmer Name | Animal No | Species | Colour | Sex | Age | Weight Kg | PCV% | Buffy Coat | Thick Smear | Thin Smear | Remarks/Observations |
| --- | --- | --- | --- | --- | --- | --- | --- | --- | --- | --- | --- | --- |
| 1 |  |  |  |  |  |  |  |  |  |  |  |  |
| 2 |  |  |  |  |  |  |  |  |  |  |  |  |
| 3 |  |  |  |  |  |  |  |  |  |  |  |  |
| 4 |  |  |  |  |  |  |  |  |  |  |  |  |
| 5 |  |  |  |  |  |  |  |  |  |  |  |  |
| 6 |  |  |  |  |  |  |  |  |  |  |  |  |
| 7 |  |  |  |  |  |  |  |  |  |  |  |  |
| 8 |  |  |  |  |  |  |  |  |  |  |  |  |
| 9 |  |  |  |  |  |  |  |  |  |  |  |  |
| 0 |  |  |  |  |  |  |  |  |  |  |  |  |
| 1 |  |  |  |  |  |  |  |  |  |  |  |  |
| 2 |  |  |  |  |  |  |  |  |  |  |  |  |
| 3 |  |  |  |  |  |  |  |  |  |  |  |  |
| 4 |  |  |  |  |  |  |  |  |  |  |  |  |
| 5 |  |  |  |  |  |  |  |  |  |  |  |  |
| 6 |  |  |  |  |  |  |  |  |  |  |  |  |
| 7 |  |  |  |  |  |  |  |  |  |  |  |  |
| 8 |  |  |  |  |  |  |  |  |  |  |  |  |
| 9 |  |  |  |  |  |  |  |  |  |  |  |  |
| 0 |  |  |  |  |  |  |  |  |  |  |  |  |

Mean PCV Not Affected: ____ Mean PCV Affected: _____ Mean PCV: ______ Total No. Positives: _____ Prevalence/Incidence: _______________

Sheet __of__

**Trypanosomosis Survey/Surveillance Summary Sheet**

Month/Year: ___________________/_____________ Province: ______________________ District: ____________________________

Date from: ________________ to _________________(DD-MM) Recorder: ______________________

| Village/Crush pen name | Longitude (E) | Latitude (S) | Elevation m | Date | Sampling frame | No. of animal presented | No. of animals sampled | %  Mean  PCV | Buffy coat | Thick smear | Thin smear | Total No. of positives | %  Prev |
| --- | --- | --- | --- | --- | --- | --- | --- | --- | --- | --- | --- | --- | --- |
|  |  |  |  |  |  |  |  |  |  |  |  |  |  |
|  |  |  |  |  |  |  |  |  |  |  |  |  |  |
|  |  |  |  |  |  |  |  |  |  |  |  |  |  |
|  |  |  |  |  |  |  |  |  |  |  |  |  |  |
|  |  |  |  |  |  |  |  |  |  |  |  |  |  |
|  |  |  |  |  |  |  |  |  |  |  |  |  |  |
|  |  |  |  |  |  |  |  |  |  |  |  |  |  |
|  |  |  |  |  |  |  |  |  |  |  |  |  |  |
|  |  |  |  |  |  |  |  |  |  |  |  |  |  |
|  |  |  |  |  |  |  |  |  |  |  |  |  |  |
|  |  |  |  |  |  |  |  |  |  |  |  |  |  |
|  |  |  |  |  |  |  |  |  |  |  |  |  |  |

Comments: _____________________________________________________________________________________________________________________

_____________________________________________________________________________________________________________________
