# Supplementary material for: Developing a national atlas to support the progressive control of tsetse-transmitted animal trypanosomosis in Zambia
Source: Parasit Vectors. 2025 Nov 10;18:452. doi: 10.1186/s13071-025-07086-2 (PMC12604332; doi:10.1186/s13071-025-07086-2)
Supplement: Supplementary file 3 — Additional file 3: Database. Tsetse geospatial database information guide. [file 13071_2025_7086_MOESM3_ESM.docx]

**Tsetse geospatial database information guide**

This information guide describes how data on the occurrence of tsetse flies are assembled into the tsetse geospatial database within the Zambia Atlas of tsetse and animal African trypanosomiasis (AAT).

1. **Source_file_name**: Gives the file name and hyperlink to the original report or record sheet where data was obtained.
2. **Type_of_survey**: It reports the objective of the survey whether Baseline (where no control has occurred) or Monitoring (where control has been undertaken)
3. **Funding**: Provides details on the source of funds for the survey.
4. **Tsetse_intervention**: It reports whether tsetse interventions have occurred in the survey area. “Yes”, where interventions are or have previously taken place and “No”, where no intervention have been undertaken.
5. **Type_of_intervention**: It reports what type on interventions have taken place in the survey area.
6. **Tsetse_belt**: Gives the name of the tsetse belt where the survey was conducted. The first letter of the tsetse belt name should be capitalized.
7. **Province**: Gives the name of the province where the survey was conducted. The first letter of the provincial name should always be capitalized.
8. **District**: Gives the name of the district where the survey was conducted. The first letter of the district name should always be capitalized.
9. **Location_village**: Gives the name of the village where the survey was conducted. The first letter of the village name should always be capitalized.
10. **Elevation_m**: The elevation of the trap site in meters.
11. **Longitude**: Longitude of the trap site in decimal degrees (Datum: WGS84)
12. **Latitude**: Latitude of the trap site in decimal degrees (Datum: WGS84)
13. **Geo_source**: Source of geo-positioning, which may be GPS or one of the many available gazetteers such as QGIS, Google Earth, GTOPO30 digital elevation model, etc.
14. **Start_date**: Starting date of the survey.
15. **Start_month**: Starting month of the survey.
16. **Start_year**: Starting year of the survey.
17. **End_date**: Ending date of the survey.
18. **End_month**: Ending month of the survey
19. **End_year**: Ending year of the survey
20. **Duration_days**: Number of trap days a trap remained at a site.
21. **Trap_type**. The type of the trap used in the survey which maybe, Epsilon, Large Sticky Trap, Tiny Sticky Trap, or any other trap that maybe used. Naming should be consistent in the database as exemplified here.
22. **Trap_notes**: Details of the trap and strategy of trap deployment (e.g. in which habitats were traps deployed? At what hours of the day where the traps operated.
23. **Odour_attractant**: Where odour attractants used in the study? The answer should be “Yes” or “No”.
24. **Attractants_used**: If the answer in 23 is yes, state the attractants used, e.g MEK, Octenol, Cow Urine, etc.
25. **Gmm_m**: The number of male *Glossina morsitans morsitans* flies caught in the trap.
26. **Gmm_f**: The number of female *G. m. morsitans* flies caught in the trap.
27. **Gmm_Total**: The total number of *G. m. morsitans* flies caught in the trap.
28. **Gmc_m**: The number of male *G. m. centralis* flies caught in the trap.
29. **Gmc_f**: The number of female *G. m. centralis* flies caught in the trap.
30. **Gmc_Total**: The total number of *G. m. centralis* flies caught in the trap.
31. **Gp_m**: The number of male *G. pallidipes* flies caught in the trap.
32. **Gp_f**: The number of female *G. pallidipes* flies caught in the trap.
33. **Gp_Total**: The total number of *G. pallidipes* flies caught in the trap.
34. **Gb_m**: The number of male *G. brevipalpis* flies caught in the trap.
35. **Gb_f**: The number of female *G. brevipalpis* flies caught in the trap.
36. **Gb_Total**: The total number G. *brevipalpis* flies caught in the trap.
37. **Gfm_m**: The number of male *G. fuscipes martini* flies caught in the trap.
38. **Gfm_f**: The number of female *G. f. martini* flies caught in the trap.
39. **Gfm_Total**: The total number of *G. f. martini* flies caught in the trap.
40. **Total**: The combined total number of tsetse caught in the trap
41. **Tsetse_P**: Tsetse presence (Presence: Yes, Absence: No)

**For data captured using mobile sampling devices, a separate database is provided which includes the following unique columns.**

1. **Flyround_Code**: An arbitrary code used to identify a particular fly round in a location.
2. **Stop_No**: An arbitrary number used to identify a stop with a fly round a specific fly round at a location.
3. **Duration_min**: Time taken to complete 1 km of fly round
4. **Flyround_type**: Type of sampling device used in the fly round. Either Black-Screen Fly Round or Vehicle-Mounted Sticky Trap
5. **Flyround_notes**: Details of the sampling design used during the fly round, e.g. Along road, footpath, stream or transect through the bush.
6. **Stop_distance_Km**: Distance between stops. Standardised to 1 Km.

**Appendix: Tsetse belts**

1. Eastern Tsetse Belt
2. Kafue Tsetse Belt
3. Kwando-Zambezi Tsetse Belt
4. Lower-Zambezi Tsetse Belt
5. Upper-Zambezi Tsetse Belt
6. Tanganyika-Mweru Tsetse Belt
7. Bangweulu Tsetse Belt
