# Supplementary material for: Developing a national atlas to support the progressive control of tsetse-transmitted animal trypanosomosis in Zambia
Source: Parasit Vectors. 2025 Nov 10;18:452. doi: 10.1186/s13071-025-07086-2 (PMC12604332; doi:10.1186/s13071-025-07086-2)
Supplement: Supplementary file 4 — Additional file 4: Database. Animal African trypanosomiasis geospatial database information guide. [file 13071_2025_7086_MOESM4_ESM.docx]

**Animal African trypanosomiasis geospatial database information guide**

This information guide describes how data on the occurrence and prevalence of are assembled into the animal African trypanosomiasis (AAT) geospatial database within the Zambia Atlas of tsetse and AAT.

1. **Source_file_name**: Gives the file name and hyperlink to the original report or record sheet where data was obtained.
2. **Type_of_survey**: It reports the objective of the survey whether Baseline (where no control has occurred) or Monitoring (where control has been undertaken)
3. **Funding**: Provides details on the source of funds for the survey.
4. **Tsetse_intervention**: It reports whether tsetse interventions have occurred in the survey area. “Yes”, where interventions are or have previously taken place and “No”, where no intervention have been undertaken.
5. **Type_of_intervention**: It reports what type on interventions have taken place in the survey area **‘ITT’ or ‘Aerial spray’**
6. **Tsetse_belt**: Gives the name of the tsetse belt where the survey was conducted. The first letter of the tsetse belt name should be capitalized.
7. **Province**: Gives the name of the province where the survey was conducted. The first letter of the provincial name should always be capitalized.
8. **District**: Gives the name of the district where the survey was conducted. The first letter of the district name should always be capitalized.
9. **Location_village**: Gives the name of the village where the survey was conducted. The first letter of the village name should always be capitalized.
10. **Elevation_m**: The elevation of the sampling site (crush pen) in meters.
11. **Longitude**: Longitude of the sampling site (crush pen) in decimal degrees (Datum: WGS84) **‘6 decimal places’**
12. **Latitude**: Latitude of the sampling site (crush pen) in decimal degrees (Datum: WGS84) **‘6 decimal places’**
13. **Geo_source**: Source of geo-positioning, which may be GPS or one of the many available gazetteers such as QGIS, Google Earth, GTOPO30 digital elevation model, etc.
14. **Date**: Date when the survey was conducted. **Format ‘DD/MM/Year’**
15. **Month**: Month when the survey was conducted. The first letter of the **‘Month’** should be capitalized.
16. **Year**: Year when the survey was conducted. The first letter of the **‘Year’** should be capitalized.
17. **Animals_presented**: Number of animals that farmers presented during the survey.
18. **Sample_size**: Number of animals sampled.
19. **Animal_species**: The species of animal samples **‘Cattle, Goat, Dog, Donkey’.** The first letter should be capitalised.
20. **Sex**: The sex of the animals sampled
21. **Age**: The age of the animal’s samples whether “Adult” or “Young” animals.
22. **Sampling_strategy**: Describes whether a random approach was used (e.g. in a study designed to assess the general epidemiological situation in an area), or a purposeful one (e.g. where specific villages/areas/herds/animals are investigated because of some peculiar features).
23. **Chemotherapy**: Reports the use of therapeutic and/or prophylactic anti trypanosomal drugs. **‘Yes’ or ‘No’**
24. **Diagnostic**: Reports the diagnostic method used during the study.
25. **Mean_PCV_not_affected**: Average Packed-Cell-Volume for animals that did not test positive for trypanosomiasis
26. **Mean_PCV_affected**: Average Packed-Cell-Volume for animals that tested positive for trypanosomiasis.
27. **Mean_PCV**: Average Packed-Cell-Volume for the survey herd
28. **Tv**: Number of animals positive for *Trypanosoma vivax.*
29. **Tc:** Number of animals positive for *T. congolense*.
30. **Tb:** Number of animals positive for *T. brucei s.l.*
31. **Total:** Total number of animals positive (single infections).
32. **TvPR:** Prevalence (in percentage) of *T. vivax*.
33. **TcPR:** Prevalence (in percentage) of *T.* congolense.
34. **TbPR:** Prevalence (in percentage) of *T. brucei s.l.*
35. **TPR:** Total AAT Prevalence (in percentage) for all trypanosomes observed.
36. **Tv_Presence: ‘**Yes’ if *T. vivax* is present, ‘No’ if the infection is not present.
37. **Tc_Presence:** ‘Yes’ if *T. congolense* is present, ‘No’ if the infection is not present.
38. **Tb_Presence:** ‘Yes’ if *T. brucei s.l* is present, ‘No’ if the infection is not present.
39. **AAT_Presence:** ‘Yes’ if animal African trypanosomiasis is present, ‘No’ if the disease is not present.
40. **TvTc:** Number of *T. vivax* and *T.* *congolense* mixed infections
41. **TvTb:** Number of *T. vivax* and *T. brucei s.l* mixed infections
42. **TcTb:** Number of *T.* *congolense* and *T. brucei s.l* mixed infections
43. **Tv_Tc_Tb:** Number of *T. vivax, T. congolense* and *T. brucei s.l* mixed infections
44. **Tv_TcPR:** Prevalence (in percentage) of *T. vivax* and *T.* *congolense* mixed infections
45. **Tv_TbPR:** Prevalence (in percentage) of *T. vivax* and *T. brucei s.l* mixed infections
46. **Tv_Tc_TbPR:** Prevalence (in percentage) of *T. vivax, T. congolense* and *T. brucei s.l* mixed infections
47. **Tv_Tc_presence: ‘**Yes’ if *T. vivax, T. congolense* mixed infection is present, ‘No’ if the infection is not present
48. **Tv_Tb_presence: ‘**Yes’ if *T. vivax, T. brucei s.l* mixed infection is present, ‘No’ if the infection is not present
49. **Tc_Tb_presence: ‘**Yes’ if *T. congolense,* *T. brucei s.l* mixed infection is present, ‘No’ if the infection is not present.
50. **Tv_Tc_Tb_presence: ‘**Yes’ if *T. vivax, T. congolense,* *T. brucei s.l* mixed infection is present, ‘No’ if the infection is not present.

**Appendix: Tsetse belts**

1. Eastern Tsetse Belt
2. Kafue Tsetse Belt
3. Kwando-Zambezi Tsetse Belt
4. Lower-Zambezi Tsetse Belt
5. Upper-Zambezi Tsetse Belt
6. Tanganyika-Mweru Tsetse Belt
7. Bangweulu Tsetse Belt
